# Key Chagas disease missing knowledge among at-risk population in Spain affecting diagnosis and treatment

**DOI:** 10.1186/s40249-021-00841-4

**Published:** 2021-04-23

**Authors:** María Romay-Barja, Laura Iglesias-Rus, Teresa Boquete, Agustín Benito, Teresa Blasco-Hernández

**Affiliations:** 1 National Centre of Tropical Medicine, Instituto de Salud Carlos III, Madrid, Spain; 2 Collaborative Research Network on Tropical Diseases, RICET, Madrid, Spain

**Keywords:** Chagas disease, Knowledge, Barrier, Diagnosis, Treatment, Non-endemic country

## Abstract

**Background:**

Chagas disease is endemic in Latin America and, over the last few decades, due to population movements, the disease has spread to other continents. Early diagnosis and treatment are critical in terms of improving outcomes for those living with Chagas disease. However, poor knowledge and awareness is one of barriers that affects access to Chagas disease diagnosis and treatment for the population at risk. Information regarding immigrants’ knowledge concerning Chagas disease control and prevention is insufficient in non-endemic countries and, therefore, this study sought to assess Chagas disease knowledge and awareness within the Bolivian community residing in Madrid.

**Methods:**

This cross-sectional study was carried out in March–August 2017. A total of 376 Bolivians answered a structured questionnaire. A knowledge index was created based on respondents’ knowledge about transmission, symptoms, diagnosis, and place to seek treatment. Multivariate logistic regressions analyses were performed to assess the factors associated with respondents’ knowledge of Chagas disease.

**Results:**

A total 159 (42.4%) of Bolivians interviewed about their knowledge of Chagas disease were men and 217 (57.6%) were women. Vinchuca was mentioned as mode of transmission by 71% of the Bolivians surveyed, while only 9% mentioned vertical transmission. Almost half of the Bolivians did not know any symptom of Chagas disease and only 47% knew that a specific blood test is necessary for diagnosis. Most of Bolivians were aware of the severity of Chagas disease, but 45% of Bolivians said that there is no cure for Chagas and 96% did not know any treatment. Based on the index of knowledge generated, only 34% of Bolivians had a good knowledge about Chagas disease transmission, symptoms, diagnosis and treatment. According to the multiple logistic regression analysis, knowledge regarding Chagas disease, diagnosis and treatment was significantly higher amongst older Bolivians who had secondary education at least, as well as amongst those who had already been tested for Chagas disease.

**Conclusions:**

This study found that most of the Bolivian population living in Spain had poor knowledge about Chagas disease transmission, symptoms, diagnostic methods and treatment. A poor understanding of the disease transmission and management is one of the most important barriers when it comes to searching for early diagnosis and appropriate care.

**Figure:**
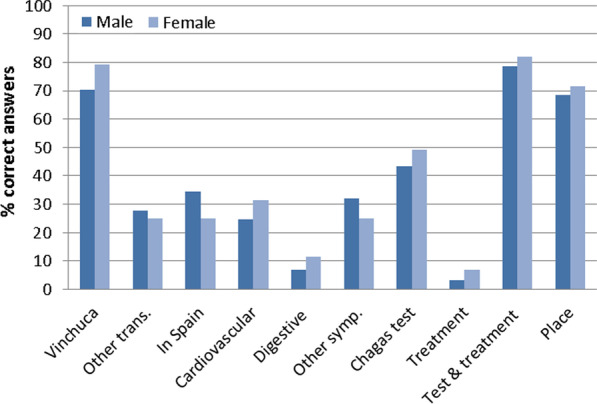

## Background

Chagas disease (CD) is a parasitic disease caused by the flagellated protozoan *Trypanosoma cruzi*. Considered a neglected tropical disease, Chagas disease is endemic in 21 countries in Latin America, where more than 6 million people are infected [1]. Over the last few decades, the disease has spread to other continents due to population movements. *T. cruzi* is transmitted to humans by triatomine bugs, popularly known as Vinchucas. Vertical transmission, blood transfusions or solid organ transplants are other routes of transmission that have a major role in non-endemic countries [2].

Chagas disease has an asymptomatic acute phase in which parasitaemia levels are higher; it then enters in an indeterminate chronic phase, with an absence of clinical symptoms of visceral involvement [3]. Up to 30% of chronically infected people develop cardiac alterations, and up to 10% develop digestive, neurological or mixed alterations which may require specific treatment. In chronic patients, antiparasitic treatment can potentially prevent or curb disease progression [1]. Recent studies have shown that treating infected women before pregnancy eliminates vertical transmission of *T. cruzi* [4, 5], whilst treatment efficacy is also especially high in congenitally infected newborns, with a cure rate of 100% [5]. The World Health Organization recently recommended implementing active strategies in Europe and other non-endemic regions in order to detect, screen and diagnose all infected pregnant women, as well as their infected newborns and siblings, and to treat them as expeditiously as possible [6].

It is estimated that the prevalence of CD amongst the Latin American population living in Europe is around 4.2% [7], and Spain is the European country with the highest burden of CD, with an estimated 52 000 cases [8].

Early diagnosis and treatment are critical in order to improve outcomes for those living with CD. However, scarce knowledge and awareness is one of the barriers that affects access to CD diagnosis and treatment for the population at risk [9], even in non-endemic countries. Misconceptions acquired in the country of origin are important barriers that prevent immigrants from undergoing screening [10]. Even when knowledge regarding CD has not always being sufficient to change care-seeking behaviour [11], awareness has emerged as an important factor for being screened [12]. Community knowledge relating to vector-borne diseases varies depending on many sociodemographic factors such as gender, department of origin, rurality and educational status that may determine participation in control activities such as screening and treatment [9, 13, 14]. Improved knowledge of CD could lead communities to adopt better health-seeking related behaviours [15]. Awareness and knowledge should be understood and addressed in terms of their relationship with socioeconomic inequalities and gaps in the public health response to CD, and cultural differences [9, 16]. Therefore, more studies are needed about the knowledge of CD among the population at risk in non-endemic countries. This study sought to assess CD knowledge and awareness within the Bolivian community living in Madrid, Spain, in order to generate useful data for the design of public and private educational campaigns regarding CD aimed at promoting Chagas screening and treatment.

## Methods

### Study area and population

This cross-sectional study was carried out in March–August 2017. The survey aimed to provide information regarding the knowledge, attitudes and practices of Bolivians living in Madrid, Spain. Based on the municipal census, a total of 15,951 Bolivians (6758 men and 9193 women) live in Madrid, distributed mainly throughout the neighbourhoods of Usera, Carabanchel, Puente de Vallecas and La Latina [17].

### Sampling and data collection

A sample size of the Bolivian population living in Madrid was calculated based on a 95% confidence level, 5% error rate, and CD knowledge rate of 50% [18, 19]. A total of 376 people were selected amongst the Bolivians found in the waiting room of the Bolivian Consulate in Madrid. A structured questionnaire was administered to participants according to the percentages of men and women in the studied population. Inclusion criteria were that the respondents had to be over 18 years of age and they have ever heard about CD (only seven interviewed stated that they have never heard of CD).

Participants were asked about their knowledge, beliefs, and attitudes related to CD. They were interviewed about their socioeconomic characteristics and knowledge related to the transmission and symptoms of CD; diagnostic methods and treatment; and their attitudes towards the stigma associated with CD. The questionnaire used was previously tested on the Bolivian population and in the same circumstances as the survey. The methodological aspects have been described elsewhere [20].

### Data analysis

A descriptive univariate analysis of participant characteristics was conducted using frequency tables for categorical variables. For normally or not-normally distributed continuous variables, we used mean and standard deviation or median and interquartile range, respectively. Differences in sociodemographic characteristics and disease knowledge and attitudes between men and women were assessed using the Chi-squared test for independence for categorical variables. For normally or not-normally distributed continuous variables, we used the Student’s *t* test or the non-parametric Mann–Whitney test, respectively. *P* values < 0.05 were considered to be statistically significant. Data analyses were performed using STATA software version 15 (StataCorp LP, Texas, USA).

In order to assess the determinants of respondents’ knowledge of CD, a knowledge index was calculated, taking into account the answers they gave to some of the survey questions [21]. In order to achieve a maximum score of 10, the respondents had to know some general facts about CD: that CD is transmitted by the Vinchuca (1 point); other correct forms of transmission (1 point); any possible form of transmission in Spain (1 point); cardiovascular disorders constitute a main symptom of CD (1 point); digestive disorders constitute a main symptom of CD (1 point); other symptoms of CD (1 point); diagnostic methods (1 point); treatments for CD (1 point); that it is possible to be tested and treated for CD in Spain (1 point); possible places where to be diagnosed and treated in Spain (1 point). Poor knowledge of CD was defined as scores either below or within the median. Scores above the median were considered as good knowledge.

The collinearity between independent variables was checked and, when present, the variable that explained the data distribution least was removed. A multiple logistic regression model was obtained using a backward stepwise procedure. The odd ratio (*OR*) and 95% confidence interval (*CI*) were computed. *P* values < 0.05 were considered statistically significant.

## Results

A total of 376 Bolivians were interviewed regarding their knowledge of CD, of which 159 (42.4%) were men and 217 (57.6%) were women. The socioeconomic and demographic characteristics of the surveyed population are summarized in Table 1. The participants had a mean age of 38 years [interquartile range (IQR): 33–45, minimum 18, maximum 77]. Most of them (76.1% of the men and 72.8% of the women) reported that they had completed secondary school studies or higher.

**Table 1:** Socio-economic and demographic characteristics of participants by sex

	Male		Female		*P-value*
	*n* = 159	%	*n* = 217	%	
Age, years					
18–24	15	9.4	14	6.5	
25–34	47	29.5	48	22.1	
35–44	56	35.2	87	40.1	
45–54	27	17.0	46	21.2	
55–64	11	6.9	12	5.5	
> 65	3	1.9	10	4.6	0.241
Education					
Primary school or less	38	23.9	59	27.2	
Secondary school or more	121	76.1	158	72.8	0.471
Bolivian department					
Cochabamba^a^	57	35.8	96	44.2	
Santa Cruz^a^	68	42.8	82	37.8	
La Paz	18	11.3	17	7.8	
Potosi	5	3.1	4	1.8	
Chuquisaca^a^	4	2.5	5	2.3	
Oruro	3	1.9	4	1.8	
Beni	1	0.6	6	2.8	
Tarija^a^	3	1.9	2	0.9	
Pando	0	0.0	1	0.5	0.469
Departments according to Chagas disease prevalence					
Non-endemic department	27	17.0	32	14.8	
Endemic department	132	83.0	185	85.3	0.327
Area					
Rural	42	26.4	58	26.7	
Urban	117	73.6	159	73.3	0.521
Madrid district					
Less than 10% of Bolivian population	57	35.8	75	34.6	
More than 10% of Bolivian population	102	64.2	142	65.4	0.440
Are you currently working?					
No	53	33.3	66	30.4	
Yes	106	66.7	151	69.6	0.548
Jobs					
Professionals, managers and technicians	5	4.7	7	4.6	
Services and sales	29	27.4	43	28.5	
Manual jobs	72	67.9	103	68.2	0.911
Household income					
None	5	3.1	7	3.2	
< EUR 1000	51	32.1	103	47.5	
EUR 1001–2000	81	50.9	86	39.6	
> EUR 2000	17	10.7	13	6.0	
Don't know	5	3.1	8	3.7	0.032
Public Health Insurance card					
No	24	15.1	25	11.5	
Yes	135	84.9	192	88.5	0.309
Received information about Chagas disease in Spain					
No	121	76.1	151	69.6	
Yes	38	23.9	66	30.4	0.163
Tested for Chagas disease					
No	96	60.4	114	52.5	
Yes	63	39.6	103	47.5	0.130

^a^Departments where Chagas disease is endemic in Bolivia

Concerning their place of origin, most of the Bolivians came from the Cochabamba and Santa Cruz departments (40.7% and 39.9%, respectively). Moreover, 73.4% of the participants were from an urban area, whilst 26.6% came from rural environments.

Regarding their settlement in Madrid, the population surveyed was distributed throughout the city’s districts in proportion to the municipal census. Most of them lived in the districts of Usera (19.4%), Carabanchel (18.6%), Puente de Vallecas (10.9%), La Latina (8.2%) and Ciudad Lineal (7.7%). With respect to their economic situation, 68.3% of the participants had a job, presenting significant differences in terms of occupation and household income between men and women. The men worked mainly in services, sales, crafts and related trade jobs (54.7%), whereas the women were more often employed in elementary occupations such as domestic cleaning or as helpers (66.9%). Amongst the men, 61.6% reported earning more than EUR 1000 as their household monthly income, whilst 50.7% of the women reported earning less.

With respect to health assistance, 87.0% of the surveyed Bolivians had the Public Health Insurance card. Moreover, 72.3% of the participants pointed out that they had not received any information about CD in Spain, and only 44.2% had been tested for CD.

### Chagas disease transmission knowledge

Regarding CD transmission (see Table 2), 75.5% of the participants knew how it is transmitted, with differences between men and women (70.4% and 79.3% respectively, *P* = 0.049). The main route of transmission identified was the Vinchuca bite (71.3%, *P* = 0.017), followed by blood transfusion (15.2%) and vertical transmission (8.8%). When asked about the possibility of CD transmission in Spain, 45.7% of Bolivians pointed out that it is possible; the main routes of transmission they identified were blood transfusion (24.2%), Vinchuca bite (16.0%), vertical transmission (6.9%) and organ transplant transmission (3.7%). Organ transplant transmission was identified more frequently by men than by women (*P* = 0.005).

**Table 2:** Bolivians’ knowledge of Chagas disease transmission by sex

	Male	%	Female		%	*P*-value
Do you know how Chagas disease is transmitted?						
Yes	112	70.4	172		79.3	
No	47	29.6	45		20.7	0.049
If so, how?^a^						
Vinchuca bite	103	92.0	165		95.9	0.017
Blood transfusion	27	24.1	30		17.4	0.399
Vertical transmission	13	11.6	20		11.6	0.725
Do you know if Chagas disease can be transmitted in Spain?						
Yes	89	56.0	83		38.3	
No	40	25.2	93		42.9	
Don’t know	30	18.9	41		18.9	0.001
If so, how?^a^						
Blood transfusion	48	53.9	43		51.8	0.020
Vinchuca bite	30	33.7	30		36.1	0.120
Vertical transmission	14	15.7	12		14.5	0.216
Organ transplant	11	12.4	3		3.6	0.005
Sexual relations	9	10.1	5		6.0	0.089
From an infected mother through breast milk?						
Yes	49	30.8	75		34.6	
No	69	43.4	80		36.9	
Don’t know	41	25.8	62		28.6	0.441

^a^Multiple choice question, answer is “yes”

In relation to the possibility of CD transmission during breastfeeding, 39.6% of the surveyed participants answered that there was no risk, whereas 33.0% considered it was possible.

### Signs and symptoms

Regarding CD attitudes, 81.9% of the participants considered CD to be a severe disease, although almost half of them did not know any symptom of CD (Table 3). Among those who affirmed to know some symptoms of CD, heart problems (28.5%) and digestive disorders (9.6%) were the most mentioned. Moreover, fever (9.6%) was more frequently mentioned as a symptom by men than by women (*P* = 0.040), while fatigue was more mentioned by women.

**Table 3:** Bolivians’ knowledge and attitudes regarding Chagas disease symptoms by sex

	Male	%	Female	%	*P*-value
Is Chagas disease a serious disease?					
Yes	126	79.3	182	83.9	
No	33	20.8	35	16.1	0.250
Chagas disease’s symptoms^a^					
Don’t know	74	46.5	96	44.2	0.658
Heart problems	39	24.5	68	31.3	0.148
Fever	21	13.2	15	6.9	0.040
Digestive disorders	11	6.9	25	11.5	0.134
Fatigue	13	8.2	22	10.1	0.518
Dizziness	14	8.8	10	4.6	0.100
Headache	10	6.3	13	6.0	0.905

^a^Multiple choice question, answer is “yes”

### Knowledge and attitudes related to Chagas diagnosis

A blood test was the first diagnostic method of CD mentioned by 78.7% of the surveyed participants (Table 4). Amongst these, 46.8% answered that it has to be a specific requested blood analysis. Asked about whether a CD test could be carried out in Spain, most of the population (80.5%) knew that this opportunity exists. Regarding the place where they might go to be diagnosed, hospital (48.8%) was the first health institution mentioned, followed by health centres (31.7%). Community campaigns and blood banks were mentioned less.

**Table 4:** Bolivians’ knowledge and attitudes regarding Chagas disease diagnosis by sex

	Male	%	Female	%	*P*-value
Do you know what test is necessary to perform when a person has Chagas disease?^a^					
Blood test					
Blood specific analysis	69	56.1	107	61.8	
Blood routine analysis	53	43.1	65	37.6	0.337
Don’t know	34	21.4	40	18.4	0.513
Xenodiagnosis	2	1.3	5	2.3	0.370
Do you know if you can do a Chagas disease test in Spain?					
Yes	125	78.6	178	82.0	
No	2	1.3	3	1.4	
Don’t know	32	20.1	36	16.6	0.678
If so, where?					
Hospital	55	44.0	93	52.3	
Health Centre	47	37.6	49	27.5	
Don’t know	14	11.2	19	10.7	
Community campaigns	6	4.8	10	5.6	
Blood bank	3	2.4	7	3.9	0.397
Do you think doctors in Spain are familiar with Chagas disease?					
Yes	92	57.9	109	50.2	
No	31	19.5	43	19.8	
Most are not	27	17.0	48	22.1	
Don’t know	9	5.7	17	7.8	0.416
How much confidence do you have in Spanish doctors to diagnose and treat Chagas disease?					
A lot of confidence	52	32.7	81	37.3	
Quite a lot of confidence	35	22.0	41	18.9	
Some confidence	30	18.9	37	17.1	
Little confidence	31	19.5	46	21.2	
No confidence at all	11	6.9	12	5.5	0.812
Do you think you might have Chagas disease?					
No	104	65.4	117	53.9	
Yes	46	28.9	76	35.0	
Don’t know	9	5.7	24	11.1	0.046
If a mother knows she has Chagas disease, should her children be tested?					
Yes	154	96.9	212	97.7	
No	2	1.3	4	1.8	
Don’t know	3	1.9	1	0.5	0.376
And if a father knows he has Chagas disease, should his children be tested?					
Yes	143	89.9	184	84.8	
No	12	7.6	22	10.1	
Don’t know	4	2.5	11	5.1	0.292

^a^Multiple choice question, answer is “yes”

Regarding confidence in health care, half of the Bolivians interviewed (53.5%) believed that Spanish doctors are familiar with CD, with 55.6% of the respondents showing a high level of confidence in these professionals when it comes to diagnosing and treating this disease. Asked about the need to perform a CD test amongst children of positive parents, 97.3% of the Bolivians surveyed highlighted the importance of screening children if the mother has a positive diagnosis, whilst another 86.9% claimed that screening was also necessary if the father has a positive diagnosis.

Regarding the possibility of having CD, most of the Bolivians interviewed (65.4% of the men and 53.9% of the women) did not believe they were infected.

### Treatment knowledge and attitudes

Regarding the possibility of cure CD (see Table 5), 45.2% of the Bolivians declared that there is no cure for Chagas disease and 96.0% did not know what the treatment for CD might be. Among those who said to know about a treatment, benznidazole was the most frequently mentioned (93.3%). Although 80.6% of the Bolivians knew that it is possible to be treated in Spain and mentioned hospitals as the place to go for treatment (36.6%), almost 39.2% did not know where to go for treatment.

**Table 5:** Bolivians’ knowledge and attitudes regarding Chagas disease treatment by sex

	Male	%	Female	%	*P*-value
Can Chagas disease be cured?					
Yes	51	32.1	64	29.5	
No	69	43.4	101	46.5	
Don’t know	35	22.0	38	17.5	
Depends on the progress	4	2.5	13	6.0	
Yes, in children under 1 year old	0	0.0	1	0.5	0.338
Do you know what the treatment for Chagas disease is?					
Yes	5	3.1	10	4.6	
No	154	96.9	207	95.4	0.473
Do you know if Chagas disease can be treated in Spain?					
Yes	125	78.6	178	82.0	
No, not in Spain	34	21.4	39	18.0	0.409
If so, where?					
Hospital	49	39.2	62	34.8	
Health centre	35	28.0	34	19.1	
Don’t know	37	29.6	71	39.9	
Other	2	0.8	2	0.0	0.163
If you had Chagas disease, would you take the treatment?					
Yes	156	98.1	208	95.9	
No	3	1.9	9	4.2	0.218
And if it were your child who had Chagas disease, would you treat him/her?					
Yes	158	99.4	215	99.1	
No	1	0.6	2	0.9	0.753
Can a pregnant woman receive Chagas disease treatment during pregnancy?					
Yes	16	10.1	18	8.3	
No	93	58.5	135	62.2	
Don’t know	50	31.5	64	29.5	0.725

Asked about the administration of treatment, most of the surveyed population (96.8%) said that they would take it. Furthermore, 99.2% of the Bolivians stated that they would treat their children if positive. In the case of pregnant women, 60.6% of the participants stated that it is not possible to be treated during pregnancy, whilst 30.3% did not know whether it is safe or not.

### Attitudes towards stigma

With regard to stigma (see Table 6), 56.6% of the participants considered that the probability of having CD was higher amongst people who lived in the countryside. Furthermore, poor people (14.1%), residents in the warmer parts of Bolivia (10.9%) and those living in adobe houses (10.1%) were other factors mentioned.

**Table 6:** Bolivians’ attitudes regarding Chagas disease stigma by sex

	Male	%	Female	%	*P*-value
What kind of people do you think are most likely to have Chagas disease?^a^					
People from the countryside	85	53.5	128	59.0	0.285
Poor people	21	13.2	32	14.7	0.672
All people equally	15	9.4	31	14.3	0.156
People from the warm parts of Bolivia	21	13.2	20	9.2	0.22
People living in adobe houses	12	7.6	26	12.0	0.107
People living in small villages	19	11.9	12	5.5	0.025
Elderly people	9	5.7	2	0.9	0.007
Do you believe that people with Chagas disease are socially rejected in Spain?					
Yes	35	22.0	45	20.7	
No	94	59.1	130	59.9	
Don’t know	30	18.9	42	19.4	0.955
If not, why?					
CD is not contagious	33	35.1	60	46.2	
People in Spain don’t know about CD	22	23.4	16	12.3	
It’s a disease without symptoms	7	7.4	19	14.6	
I haven't heard of any cases	9	9.6	13	10.0	0.052
If you had Chagas disease, who would you tell?^a^					
The whole family	64	40.3	101	46.5	0.225
My partner	88	55.3	59	27.2	0.000
Parents	32	20.1	39	18.0	0.598
Children	21	13.2	37	17.0	0.308
Medical practitioners	9	5.7	20	9.2	0.202
Friends	14	8.8	12	5.5	0.216

^a^Multiple choice question, answer is “yes”

In relation to the social rejection of people with CD in Spain, 59.6% of the surveyed population stated that this was not a common experience. The reasons given to justify the absence of stigma in Spain were that the disease is not contagious (41.5%), and lack of knowledge about CD amongst Spanish people (16.9%).

Asked about who they would tell if they were diagnosed with CD, the participants mentioned their closest social circle, mainly their family (43.9%) and their partner (39.1%), with the women being more reluctant (*P* = 0.0001). Only 15.4% would inform their children. The possibility of informing their boss or colleagues was rarely considered.

### Knowledge index

**Fig. 1:**
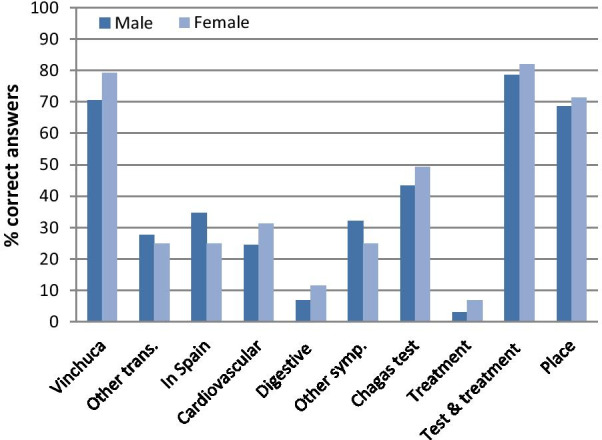
Bolivians knowledge index about Chagas disease according to sex. Vinchuca: Chagas is transmitted by the Vinchuca; Other trans.: other correct forms of transmission; In Spain: Forms of transmission in Spain; Cardiovascular: cardiovascular disorders constitute a main symptom of Chagas; Digestive: digestive disorders constitute a main symptom of Chagas; Other symp.: other symptoms of Chagas; Chagas Test: actual diagnostic methods; Treatment: Nifurtimox or Benznidazole are the treatments for Chagas; Test and treatment: to know that in Spain it is possible to be tested and treated for Chagas; Place: places where it is possible to be diagnosed and treated in Spain

The majority of surveyed Bolivians (263, 63.6%) had a poor knowledge about CD, with none of the 376 participants achieving the maximum score of 10 points. The median score was 4, without significant differences between men (66.0%) and women (61.7%) regarding this knowledge index. However, the women’s knowledge about transmission, symptoms, diagnostic testing, treatment and adequate places of diagnosis was higher than that of men (Fig. 1).

### Factors associated with Chagas disease knowledge

According to the multiple logistic regression analysis (see Table 7), knowledge about CD, diagnosis and treatment was significantly better amongst older Bolivians who possessed at least secondary education, and amongst those who had been tested for CD. A better knowledge of CD was also related to the respondents’ perception of the severity of the disease. Other variables such as department of origin, years living in Spain, district and income were not associated with CD knowledge index.

## Discussion

This study found that most of the Bolivian population living in Spain had poor knowledge about CD transmission, symptoms, diagnostic methods and treatment, with CD knowledge being better among women. A poor understanding of CD is one of the most important barriers when it comes to searching for appropriate care, especially in a non-endemic country where there are no general health education campaigns regarding Chagas disease (Table 7).

**Table 7:** Factors associated with Chagas disease Knowledge by multiple logistic regression

	Unadjusted *OR* (95% *CI*)^a^	Adjusted *OR* (95% *CI*)^a^
Age		
< 40 years	1	1
41 years or more	1.88 (1.22–2.90)	1.9 (1.18–3.05)
Education		
Primary school or less	1	1
Secondary school or more	1.58 (0.96–2.60)	2.2 (1.27–3.89)
Chagas disease is a severe disease		
No	1	1
Yes	2.56 (1.36–4.81)	2.2 (1.11–4.21)
Received information		
No	1	
Yes	2.08 (1.31–3.30)	
Tested for Chagas disease		
No	1	1
Yes	3.52 (2.27–5.47)	3.69 (2.32–5.87)

*OR*: odd ratio; *CI*: confidence interval

^a^Only for the variables that remain in the model

Serious misconceptions regarding Chagas disease transmission emerged in this study. Bolivians mostly associated the transmission of CD with the Vinchuca, and some even mentioned the Vinchuca as a possible means of transmission in Spain. This representation of CD transmission is strongly linked with the country of origin [12, 22], where the populations at risk are largely aware of the relationship between this vector and the disease [14]. However, less than half of the Bolivians surveyed seemed to be aware about the possible transmission of CD in a non-endemic country such as Spain, and while vertical transmission was known, this was spontaneously mentioned by only 7% of participants. Misunderstandings about how transmission works were also found, with most of the Bolivians responding that it was necessary to test a child if either the father or mother were positive. Furthermore, some Bolivians answered that CD could be transmitted through breast-feeding. Uncertainties about the transmission of CD amongst migrant populations at risk living in Spain have been reported before [12, 23, 24]. Unfortunately, CD transmission knowledge does not seem to have improved over time. Increasing awareness regarding vertical transmission is crucial if the objective is to improve screening and treatment rates [6].

Most of the Bolivians living in Madrid were aware of the severity of Chagas disease. However, the symptoms of the disease were unknown to almost half of the respondents. Although awareness of the disease’s severity is not common in other studies [9, 22], lack of knowledge regarding CD signs and symptoms is common [9, 12, 24, 25]. Chagas disease is a silent disease, one that is mostly asymptomatic during the indeterminate chronic phase, which makes it difficult to identify signs and later complications. The long period without symptoms that might affect everyday activities, added to the normalization of CD, often keeps patients from being diagnosed and treated [26, 27].

Conversely, the Bolivians surveyed seemed to have a good knowledge of the Spanish health system and knew that it is possible to be tested and treated in Madrid. This could be linked to the number of years they have lived in Spain. The degree of confidence they have in Spanish doctors is also quite remarkable, even when almost 40% still doubt that doctors in Spain really know about CD. However, the majority of the Bolivians living in Madrid did not believe that they had CD, even when only 44% had been tested [28]. Diagnosis of CD produces a range of reactions in individuals, from scepticism to fear and anxiety [29]. It seems that the idea that “it is better not to know” found in other studies [12] reinforces the lack of interest in knowing more about the disease. Furthermore, most of the Bolivians knew that a blood analysis was necessary to know whether they were positive with CD, although almost 32% believed that this could be known with a routine analysis and, therefore, they did not ask their doctors for a specific diagnosis. Unfortunately, most non-endemic country health systems do not promote CD screening, because practitioners are not aware of CD, vertical transmission or the benefits of prompt diagnosis [9, 30]. Improving awareness within the population at risk and amongst health workers has become a priority in order to boost diagnosis and treatment.

The idea that CD has no cure is still deeply rooted amongst the Bolivians interviewed and only one woman mentioned that CD could be cured in children under one year of age. This widespread perception amongst the population at risk reinforces the idea that there is little point in diagnosing and treating the disease [23]. However, although there is confusion regarding treatment, they at least know that treatment is available [12, 27]. What is more, most of the Bolivians interviewed had a positive attitude towards being treated and towards treat their children. This attitude is present in various studies regarding the at-risk population living in non-endemic countries [22], although, unfortunately, this is not always consistent with their behaviour in terms of healthcare. Previous studies have shown that more than half of CD-positive Bolivians living in Madrid did not begin or complete treatment [28]. However, such positive attitudes towards treatment should be reinforced by improving their knowledge about the effectiveness of treatment at the disease’s different stages.

Bolivians associated CD with poor people living in the rural areas of their country, but, at the same time, they did not believe that people with CD are rejected in Spain, mainly because it is not a contagious disease and almost nobody knows about the disease here. Although this might indicate that the perception of stigma has decreased in relation to the findings in other studies [27, 29, 31], almost none of the Bolivians mentioned their boss or colleagues when asked about whom they would tell if they had CD.

The factors associated with the Bolivians’ knowledge index of CD consisted of age, education, awareness about the severity of the disease, and whether they had been tested for CD. Young Bolivians had a poorest knowledge regarding CD transmission, diagnosis and treatment. This association between poor knowledge of CD and Bolivians’ age has been found in other regions of Spain [24], and this has become a serious problem if the aim is to treat women of childbearing age and young children [6].

Level of education and having been screened for CD are other factors associated with a better knowledge of the disease. It is clear that it is important to improve and extend educational campaigns in Spain by targeting the most vulnerable population at risk, through the existing specialised local associations and non-governmental organizations [32]. They must address the principal gaps in knowledge, especially those regarding transmission, diagnosis and treatment. Other studies have found that gaps in the at-risk population’s knowledge of CD are not only linked to their level of education, but may also be explained by doctor-patient communication [12]. Bolivians already screened for CD have a better knowledge because they have already received information about the disease from their doctors. Therefore, also improving the awareness of healthcare workers is essential so that they can provide adequate information to the population at risk [30]. A comprehensive approach is essential if we are to go beyond the biomedical aspects and address the multidimensional nature of the issue, while promoting an educational approach that allows individuals and communities to analyse and lead contextualised prevention and health promotion initiatives [33].

This study has certain limitations. First, it consisted of a cross-sectional study, so its findings may not be generally applicable to very different contexts. Second, people who answer that they had never heard of Chagas disease were not included in this study, meaning that information about some of the people at greatest risk might be excluded, despite the small number they represented. Third, due to the sample size, some associations may not show significance in the multivariable logistic regression.

## Conclusions

In order to overcome existing barriers regarding diagnosis and treatment, it would be helpful to improve knowledge and understanding regarding CD transmission and treatment. There is a need for participative educational programmes in non-endemic countries that would focus on certain key concepts:

- CD transmission occurs in non-endemic countries, mainly through vertical transmission;
- Prompt diagnosis and treatment of infected women before pregnancy could avoid vertical transmission;
- Existing treatments of Chagas cure the disease in children under the age of one and improve the prognosis in adults.

These educational campaigns should mainly target the young population at risk and primary health workers.

## Data Availability

Al data generated or analyzed during this study are included in this published article.

## Abbreviations

CD: Chagas disease
*CI*: Confidence interval
EUR: Euro
IQR: Interquartile range
*OR*: Odd ratio
USA: United States of America
